# Enhancing open-surgery gesture recognition using 3D pose estimation

**DOI:** 10.1007/s11548-025-03564-1

**Published:** 2026-01-14

**Authors:** Ori Meiraz, Shlomi Laufer, Robert Spector, Itay Or, Gil Bolotin, Tom Friedman

**Affiliations:** 1 Faculty of Data and Decision Sciences, Technion - Israel Institute of Technology, Technion City, 32000 Haifa, Israel; 2 Department of Cardiology, Rambam Health Care Campus, HaAliya HaShniya, 3109601 Haifa, Israel

**Keywords:** Gesture recognition, Action recognition, Pose estimation, Open surgery

## Abstract

**Purpose** Surgical gestures are fundamental components of surgical procedures, encompassing actions such as cutting, suturing, and knot-tying. Gesture recognition plays a pivotal role in the automatic analysis of surgical data. Although recent advancements have improved surgical gesture recognition, much of the existing research relies on simulations or minimally invasive surgery data, failing to capture the complexities of open surgery. In this study, we introduce and employ a new open surgery dataset focused on closing incisions after saphenous vein harvesting. **Methods** Our goal is to improve gesture recognition accuracy by incorporating tool pose estimation and 3D hand pose predictions of surgeons. We employ MS-TCN++  and LTContext  for gesture recognition, and further enhance performance through an ensemble of models using different modalities–video, tool pose, and hand pose data.

**Results** The results reveal that using an ensemble model combining all three modalities provides a substantial improvement over video-only approaches, leading to statistically significant gains across multiple evaluation metrics. We further demonstrate that the model can rely solely on hand and tool poses, completely discarding the video input, while still achieving comparable performance. Additionally, we show that an ensemble model using only hand and tool poses produces results that are either: statistically significantly better than using video alone, or not statistically significantly different.

**Conclusion** This study highlights the effectiveness of integrating multimodal data for surgical gesture recognition. By combining video, hand pose, and tool pose information, our approach achieves higher accuracy and robustness compared to video-only methods. Moreover, the comparable performance of pose-only models suggests a promising, privacy-preserving alternative for surgical data analysis.

## Introduction

Surgical gestures are essential components of surgical procedures, encompassing actions such as cutting, suturing, and knot-tying. Gesture recognition plays a pivotal role in the automatic analysis of surgical data. It facilitates workflow detection, identifies actions that exceed expected durations [4], and serves as a metric for assessing surgeon skill levels [23]. Extensive research has been conducted on gesture recognition within surgical simulators [20], and there are multiple datasets for minimally invasive surgery in the operating room. Despite these advances, there remains a significant research gap on gesture recognition in the context of open surgery within a surgical theater.

Open surgery videos present substantial challenges stemming from the limited control over operating room conditions, including lighting, surgeon movements, and camera placement [24]. Consequently, developing a reliable and accurate gesture recognition model becomes a particularly daunting task.

One potential approach to overcoming these challenges involves integrating multiple modalities that capture critical information beyond what current pipelines and models typically learn. With advancements in 3D computer vision, it can now be leveraged to significantly enhance gesture recognition.

Recent studies have shown the effectiveness of utilizing skeletal representations in various domains. These include providing feedback in surgical training [4], recognizing actions [6, 7], and enhancing gesture recognition [10].

In this study, we assess the combination of three modalities: RGB data, hand pose estimation, and surgical tool pose estimation. We aim to demonstrate how this multi-modal approach enhances gesture recognition accuracy. To evaluate our approach, we will introduce a new open surgery dataset focused on closing the incision following saphenous vein harvesting.

**Fig. 1:**
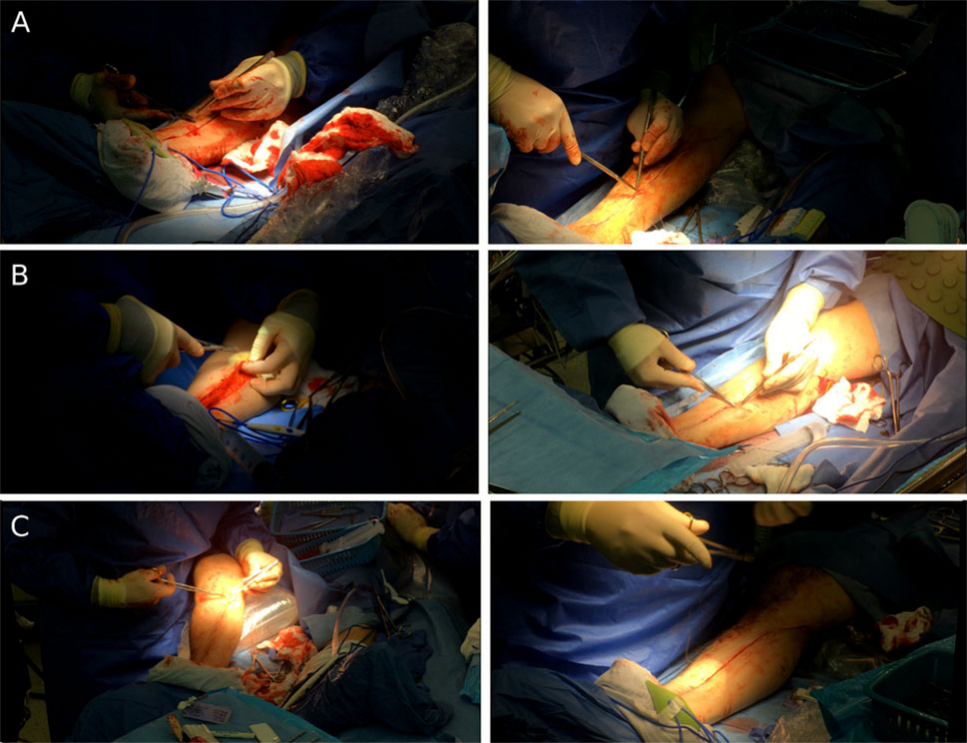
**A** A comparison between two frames from different viewing angles and distances. The image on the left has a flatter angle and is closer while the right views the leg from above and further away. **B** A comparison between two frames with different lighting

## Related work

Action segmentation predicts temporal boundaries and labels of actions in videos, with gesture recognition focusing on finer, shorter actions. Recent methods leverage Temporal Convolutional Networks (TCNs) and attention mechanisms for improved accuracy.

TCNs are fundamental in action segmentation. ED-TCN [17] introduced a hierarchical temporal structure for improved boundary detection. MS-TCN and MS-TCN++  [18, 34] refine predictions iteratively to mitigate over-segmentation issues. Self-attention mechanisms have been integrated into action segmentation to model long-range dependencies. ASFormer [32] uses self-attention across temporal segments to improve segmentation performance. Similarly, TimesFormer [3], originally designed for video action recognition, has been adapted for action segmentation, often in combination with TCNs to leverage both efficiency and temporal modeling capabilities. LTContext  [2] further improves long-term dependencies through hierarchical TCNs and contextual attention, balancing fine-grained boundary detection with overall coherence.

Lea [16] implemented a Skip-Chain Conditional Random Field to segment and recognize fine-grained activities in surgical training. Goldbraikh *et al*.[13] introduced Bounded Future MS-TCN++  to enhance segmentation by considering the future context of each action. De Rossi *et al*.[9] applied temporal convolutional networks combined with RGB and Motion History Images [1] to integrate spatial-kinematic data. ASTCFormer [33] combined temporal convolution networks with ASFormer encoders and decoders to achieve state-of-the-art results on the Cholec80 dataset [29]. TeCNO [8] fine-tuned a ResNet50 [15] feature extractor with MS-TCN [34] for segmentation. Although previous research, such as [20], has focused on open surgeries, it predominantly relied on simulated organs connected to a surgical manikin, thus representing simulated open surgery environments. Most datasets previously utilized originate from simulations [9, 12, 13, 16, 20] or minimally invasive surgeries [8, 29, 33], not actual open surgeries. Hamoud *et al*.[14] used human pose estimation from multi-view datasets, captured either in a simulation [21] or a robot-assisted surgery [26]. To the best of our knowledge, our work is the first to conduct gesture recognition on real open surgery data performed without robot assistance or a multi-view system.

The standard pipeline for action segmentation consists of two stages:

1. fine-tuning a pre-trained per-frame feature extractor.
2. Applying a temporal segmentation model on the extracted features [2, 3, 17, 18, 32, 34] .

## Dataset

Our dataset consists of 13videos executed by 5different surgeons, documenting the closure of the epidermis following saphenous vein harvesting. These videos are recorded at 30 FPS, with an average of 22509frames per video and a standard deviation of 9833, totaling approximately 162 min of footage, or an average of 12.5 min per video.

During the procedure, three primary gestures are performed: inserting the needle into the tissue using the needle driver, gripping the needle with tweezers, and passing the needle from the tweezers back to the tissue using the needle driver. Additionally, surgeons occasionally engage in actions unrelated to the suturing process, such as cleaning the tissue or pausing for another procedure to conclude. As a result, we have identified four distinct gesture classes: ’entering,’ ’gripping,’ ’passing,’ and ’other.’

In total, our dataset includes 850 ’entering’ gestures, 817 ’gripping’ gestures, 814 ’passing’ gestures, and 259 ’other’ gestures, with average lengths of 138, 41, 66, and 259 frames respectively. The videos were annotated using the Behavioral Observation Research Interactive Software (BORIS) [11].

Due to the nature of the videos, unlike in simulations, we have limited control over room conditions and face constraints with camera positioning, resulting in significant variance across the videos. The primary differences are observed in camera angle (Fig. 1A), lighting (Fig. 1B) and distance from the leg (Fig. 1C). These variations are influenced by several factors within the operating room environment, including areas that must remain sterile, feasible locations for camera placement, movements of the surgical bed, and the preferred lighting settings during operations.

## Method

The commonly used approach for action segmentation and gesture recognition primarily relies on features extracted directly from video frames. To enhance our model, we incorporate the predicted poses of the surgeon’s tools and hands. We propose two distinct approaches to effectively integrate this pose information with the extracted features:

1. A straightforward  method.
2. An ensemble of models, each relying on a different set of features.

These methods will be described in Sect. 4.3

### Feature extraction of videos

We fine-tuned a frame-wise 2D EfficientNetV2 [28] with a $224\times 224$ resolution, a standard resolution in the domain [5, 13]. For each epoch, we sampled 400samples per class and applied color jitter, Gaussian blur, and random sharpness adjustments. The model was trained for 100epochs with early stopping (patience: 5), using Adam optimization and cross-entropy loss. We experimented with small, medium, and large EfficientNetV2 models and learning rates of $10^{-3}$, $10^{-4}$, and $10^{-5}$.

### Features from pose

#### Tools pose

**Fig. 2:**
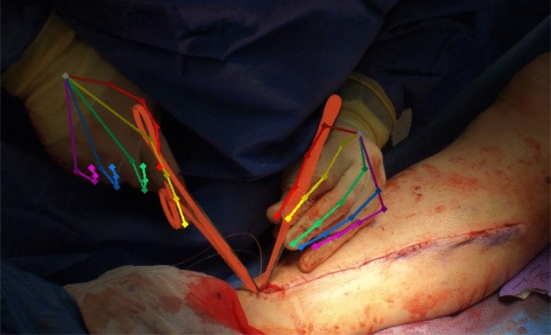
Visualization of the predicted pose for the tools (in red) and the joints

We used the algorithm presented in [27] to predict a 6 degrees-of-freedom pose (*x*, *y*, *z* location and rotation) for each tool in every frame. However, due to tool symmetry, occasional 180-degree flips around the y-axis – or simultaneous 180-degree flips around both the x- and z-axes – occurred between consecutive frames. Although these flips represent the same physical pose, they introduced temporal inconsistencies. To address this, we implemented a correction mechanism that selects the rotation minimizing the transformation from the previous pose, ensuring consistent pose representations and preventing ambiguities from propagating to downstream models. Then we applied the Last Observation Carried Forward (LOCF) imputation [19] for up to 10 frames from a detected tool and a Savitzky-Golay smoothing [25]. For model training, we augmented the tool’s pose in the following ways: 1) a random rotation of 180 degrees around the y-axis was applied to all positions in the video; 2) a random rotation of 180 degrees around both the x- and z-axes was applied to all positions in the video; and 3) random noise was added to each entry (Fig. 2).

#### Hands pose

We utilized the HaMeR  model [22] to capture the joints in each hand of the surgeon, representing the joints as an ordered set of 21 3D points. This resulted in a feature vector of size $2 \times 21 \times 3$. We chose HaMeR  for its demonstrated robustness [22]. To address frame-to-frame continuity, we applied linear interpolation for gaps of up to 10 frames and employed Savitzky-Golay smoothing to refine the data. During training, we augmented the poses with a random rotation where the rotation was applied to all joints in the video.

**Fig. 3:**
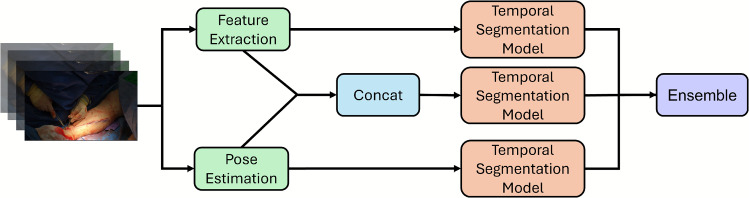
Visualization of the method. For a given video, we extract the features of the different modalities *e*.*g*. video and tools’ pose. We train a model for each modality as well as a model for the concatenated data. Finally, we ensemble the outputs

### Segmentation model

Building on previous research in the surgical field [8, 13], we employed the MS-TCN++  [18] as our primary segmentation model. Additionally, to validate our results against newer and more advanced models, we utilized the LTContext  model [2]. Given the extensive length of our videos and the significant memory requirements of the LTContext  model, we sampled every 2nd frame.

#### Straightforward  approach

Given one or more modalities, such as video and tool pose, we concatenate their features into a single feature vector per frame. We then train a gesture recognition model using this concatenated vector. We use the loss from [34] as it is a common loss for this task, used in other works [2, 13, 18]. We evaluated the effect of input normalization when using two or more modalities before inputting it to the model, with the goal of ensuring consistent feature scaling across modalities. Specifically, we applied 1D batch normalization to the input features. By comparing the results of the MS-TCN++  model with and without normalization (Tables 1 and 8), as well as the corresponding results for the LTContext  model (Tables 2 and 9), we observe that in most multi-modal settings, performance is higher without normalization. Consequently, we did not apply input normalization in our approach or in the proposed ensemble (Sect. 4.3.2).

#### Ensemble approach

**Fig. 4:**
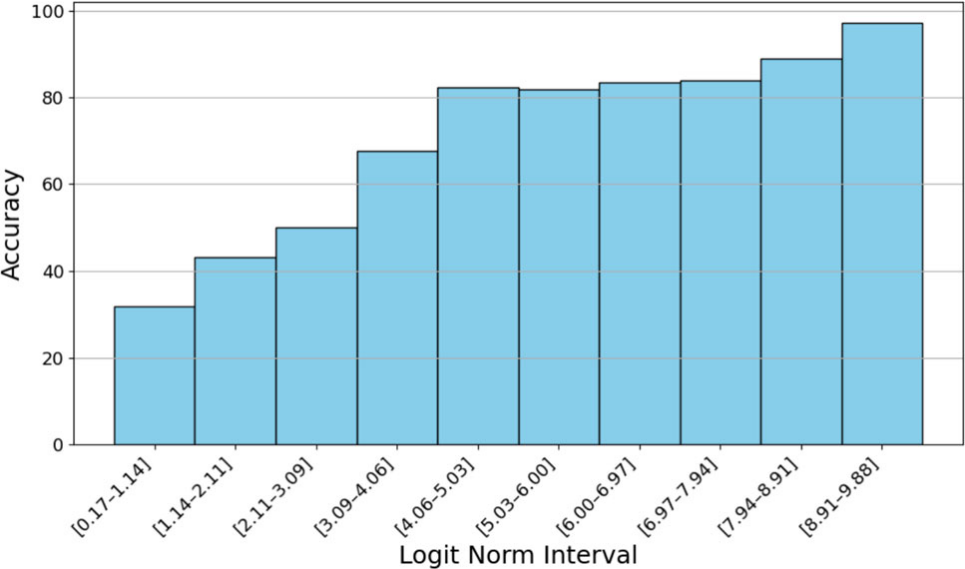
Relationship between logit norm and model accuracy. Logits are obtained from a model trained on video input. For each split, frames from the corresponding test set are used. Logit norms are grouped into 10 equal-sized bins

**Fig. 5:**
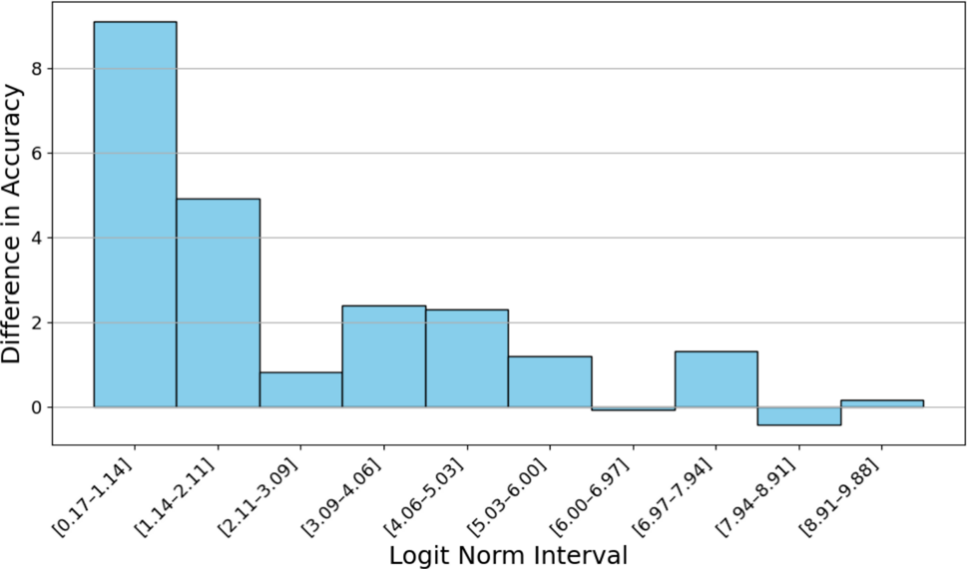
Relationship between logit norm and difference in accuracy between the ensemble model and the straightforward  method. Logits are obtained in the same way as Fig. 4

We ensemble the outputs of the models for each modality combination (e.g., video, pose, and both) which we train separately using the straightforward approach mentioned above. Wei *et al*.[30] demonstrated how confident models tend to have logits with a higher norm. So, we use the logits instead of the probabilities by averaging the logits for each frame. Thus, when a model is confident, the logits of the other models will have less effect on the final input, compared to averaging the probabilities. We demonstrate in Fig. 4 that predictions tend to be more accurate when the logits have higher norms. Furthermore, Fig. 5 shows that ensembling is most beneficial when the logits have lower norms, reflecting lower model confidence. While each model is temporally smooth due to the loss used [34], averaging the outputs may introduce temporal noise; hence, we replace the predictions of frames that differ from their neighbors with those of the surrounding frames, ensuring smoother transitions and reducing abrupt changes in the output. A visualization is provided in Fig. 3.

## Experiments

We employed a leave-one-user-out (LOUO) cross-validation scheme. In each fold, videos from one surgeon were placed in the validation set, while videos from another surgeon were assigned to the test set, with all remaining videos forming the training set. We adopted this approach to avoid training and validating on data from the same surgeon, thereby reducing the risk of overfitting. In total, there are $5\cdot (5- 1) = 20$ different folds. The reported mean and standard deviation for each metric are computed across all folds.

For the MS-TCN++  model, we set the number of layers in the refinement and prediction generation to be equal *i*.*e*., $L_{PG} = L_R = L$. For both models, we experimented on $L\in \{3, 5, 8, 12\}$ and the number of stages $S\in \{2, 3, 5\}$ with a learning rate $lr\in \{10^{-3}, 10^{-4}, 10^{-5}\}$. In LTContext  , we set the window size to 64, as done in the original paper and experimented with and without using the instance normalization. As there is a relatively small number of videos, we opted for smaller models and indeed the biggest model was rarely the best one in our experiments.

While the final hyper-parameters for a given model (e.g., MS-TCN++  ) are kept consistent across all cross-validation folds for a specific input modality (e.g., hand pose), they are not necessarily identical for a different model (e.g., LTContext  ) or the same model with a different input modality. This ensures that the final reported performance for each combination is based on its individually optimized settings. The final hyper-parameters are reported in Tables 6 and 7 in the appendix.

We used an A100 GPU and Intel(R) Core(TM) i9–10940X CPU.

**Table 1:** Metrics using MS-TCN++

	Accuracy	Edit	F1-macro	F1@10	F1@25	F1@50
Hands	72.8 ± 6.5	74.3 ± 6.1	69.5 ± 7.2	77.4 ± 6.2	73.3 ± 6.9	61.2 ± 9.9
Tools	64.5 ± 3.9	68.6 ± 3.4	60.2 ± 3.6	71.2 ± 7.1	66.1 ± 7.4	47.3 ± 7.0
Video	74.0 ± 4.8	75.4 ± 4.6	69.7 ± 6.3	79.9 ± 6.3	76.0 ± 6.6	64.3 ± 7.6
Hands & tools	67.6 ± 3.5	70.8 ± 2.9	63.2 ± 4.2	73.7 ± 8.1	69.3 ± 8.1	52.3 ± 7.5
Ensemble	75.3 ± 3.5	76.9 ± 3.0	71.8 ± 3.9	81.3 ± 2.9	77.6 ± 3.4	64.9 ± 6.2
Hands & video	75.3 ± 5.7	76.5 ± 5.5	70.8 ± 7.4	77.8 ± 7.7	73.8 ± 8.0	63.6 ± 8.6
Ensemble	74.8 ± 12.9	75.6 ± 13.1	72.1 ± 15.1	80.9 ± 16.9	77.5 ± 17.6	67.7 ± 18.0
Tools & video	76.3 ± 5.8	77.9 ± 5.3	72.3 ± 7.2	82.8 ± 6.8	79.5 ± 7.4	68.2 ± 8.8
Ensemble	77.7 ± 7.2	78.8 ± 7.2	75.0 ± 8.1	86.6 ± 7.6	83.5 ± 8.9	72.1 ± 11.4
Hands & tools & video	76.4 ± 5.1	78.0 ± 4.8	72.2 ± 6.3	80.8 ± 7.3	76.8 ± 7.5	65.9 ± 8.3
Ensemble	80.6 ± 5.4	81.6 ± 5.5	78.4 ± 5.5	89.1 ± 4.7	86.7 ± 5.4	76.6 ± 8.2

**Table 2:** Metrics using LTContext

	Accuracy	Edit	F1-macro	F1@10	F1@25	F1@50
Hands	68.9 ± 6.9	71.7 ± 6.0	65.4 ± 7.0	76.4 ± 6.9	71.8 ± 7.9	58.8 ± 11.0
Tools	62.2 ± 3.9	67.4 ± 3.2	57.4 ± 4.6	70.1 ± 6.3	63.9 ± 6.8	43.4 ± 5.9
Video	74.8 ± 4.4	76.6 ± 3.9	71.3 ± 5.6	84.2 ± 5.8	80.7 ± 6.4	68.7 ± 7.4
Hands & tools	66.1 ± 5.9	69.7 ± 6.4	60.2 ± 11.0	69.7 ± 22.2	65.0 ± 21.5	49.5 ± 16.7
Ensemble	73.5 ± 4.5	75.6 ± 3.9	69.6 ± 4.2	80.8 ± 3.5	76.4 ± 3.7	62.7 ± 6.1
Hands & video	74.8 ± 5.1	76.2 ± 4.7	71.0 ± 6.4	80.0 ± 6.9	76.2 ± 7.7	65.3 ± 8.6
Ensemble	79.4 ± 6.1	80.6 ± 6.0	76.9 ± 6.7	87.5 ± 6.8	84.4 ± 7.5	74.5 ± 9.5
Tools & video	77.8 ± 4.8	79.2 ± 4.5	73.8 ± 6.0	83.1 ± 6.1	79.6 ± 6.9	68.2 ± 7.6
Ensemble	79.4 ± 4.1	80.8 ± 4.0	76.5 ± 4.8	88.1 ± 4.1	85.1 ± 5.2	74.2 ± 8.8
Hands & tools & video	78.1 ± 4.9	79.5 ± 4.6	74.3 ± 6.1	85.2 ± 5.6	82.5 ± 6.1	72.2 ± 7.7
Ensemble	82.7 ± 2.4	83.9 ± 2.2	80.1 ± 2.9	90.8 ± 3.2	88.4 ± 3.7	79.7 ± 6.0

**Table 3:** Metrics after finetuning the extractor, without temporal knowledge

Size	Accuracy	Edit	F1-macro	F1@10	F1@25	F1@50
Small	57.5 ± 6.9	59.8 ± 6.6	49.2 ± 9.2	28.6 ± 3.6	21.5 ± 3.8	13.4 ± 3.5
Medium	58.0 ± 6.2	60.2 ± 6.1	51.9 ± 8.2	30.5 ± 3.8	23.5 ± 4.2	14.6 ± 3.6
Large	62.9 ± 5.7	64.9 ± 5.4	55.6 ± 7.8	36.1 ± 3.7	29.2 ± 4.4	20.1 ± 4.6

## Results

In Tables 1 and 2, each row displays the mean and standard deviation of the evaluated metrics for different input modalities. When multiple modalities are combined, such as hand and tool poses, two rows are dedicated to these results. The first row, labeled ’Hands & Tools,’ corresponds to the straightforward approach, while the second row represents the ensemble method. It is evident that the ensemble approach consistently improves the results.

We also show a table containing the metrics after only fine-tuning the per-frame feature extractor in Table 3*i*.*e*., without a temporal model. The extractor chosen was EfficientNetV2 Large.

### Statistical significance

To compare the results between two different methods, we use the Wilcoxon signed-rank test [31], as the results are paired. Specifically, for each video and each split in which the video appears in the test set, we obtain two corresponding values–one for each method–and assess statistical significance based on these pairs. We test two different hypotheses:

1. Using the ensemble of video, hand, and tool pose yields better results than using only the video. In Table 4 we can see a strong statistical significance ”across the board”.
2. The results from using only pose with the ensemble method differ from those obtained using video alone (Table 5). A statistically significant difference is observed only when using MS-TCN++  , with results favoring the pose-based approach.

**Table 4:** Wilcoxon signed-rank test for ensemble of all VS just video

	Accuracy	Edit	F1-macro	F1@10	F1@25	F1@50
MS-TCN++	$0.0004^*$	$0.0008^*$	$0.0000^*$	$0.0000^*$	$0.0000^*$	$0.0000^*$
LTContext	$0.0000^*$	$0.0000^*$	$0.0000^*$	$0.0016^*$	$0.0008^*$	$0.0000^*$

**Table 5:** Wilcoxon signed-rank test for using just pose VS using just video

	Accuracy	Edit	F1-macro	F1@10	F1@25	F1@50
MS-TCN++	$0.0328^*$	$0.0215^*$	$0.0107^*$	0.2943	0.2162	0.4304
LTContext	0.2455	0.1231	0.5706	0.0897	0.1140	0.1429

**Table 6:** Final hyper-parameters for the MS-TCN++  model

	*L*	*S*	*lr*	Feature size
Hands	5	2	$10^{-3}$	128
Tools	8	5	$10^{-4}$	128
Video	8	3	$10^{-3}$	128
Hands & tools	8	5	$10^{-4}$	128
Hands & video	8	2	$10^{-3}$	128
Tools & video	8	5	$10^{-3}$	128
Hands & tools & video	8	2	$10^{-3}$	128

**Table 7:** Final hyper-parameters for the LTContext  model

	*L*	*S*	*lr*	Feature size	Use instance norm	Window size
Hands	8	3	$10^{-3}$	128	True	64
Tools	8	5	$10^{-4}$	128	False	64
Video	8	5	$10^{-3}$	128	True	64
Hands & tools	8	5	$10^{-3}$	128	True	64
Hands & video	8	2	$10^{-3}$	128	True	64
Tools & video	8	2	$10^{-3}$	128	True	64
Hands & tools & video	8	3	$10^{-3}$	128	True	64

**Table 8:** Metrics using MS-TCN with normalization

	Accuracy	Edit	F1-macro	F1@10	F1@25	F1@50
Hands & tools	73.2 ± 6.2	75.1 ± 5.6	69.3 ± 6.9	81.4 ± 8.1	77.7 ± 8.3	65.5 ± 11.2
Hands & video	73.8 ± 6.1	75.1 ± 5.7	69.9 ± 7.3	76.6 ± 7.6	72.1 ± 8.1	61.4 ± 8.7
Tools & video	73.1 ± 5.8	74.7 ± 5.3	68.8 ± 7.4	80.5 ± 7.4	76.5 ± 8.4	64.4 ± 9.4
Hands & tools & video	73.6 ± 5.2	74.9 ± 5.0	69.2 ± 6.7	76.3 ± 6.9	71.5 ± 7.5	60.8 ± 8.4

**Table 9:** Metrics using LTContext  with normalization

	Accuracy	Edit	F1-macro	F1@10	F1@25	F1@50
Hands & tools	61.6 ± 10.8	65.3 ± 12.3	53.4 ± 19.4	61.6 ± 31.3	57.3 ± 29.2	42.4 ± 21.9
Hands & video	75.7 ± 5.2	77.1 ± 4.8	71.8 ± 7.0	80.7 ± 6.8	77.0 ± 7.6	66.5 ± 8.9
Tools & video	77.8 ± 4.9	79.2 ± 4.5	73.9 ± 6.5	84.4 ± 6.0	81.1 ± 7.1	70.2 ± 8.7
Hands & tools & video	77.9 ± 4.8	79.3 ± 4.4	74.1 ± 6.2	85.4 ± 5.8	82.6 ± 6.7	72.5 ± 8.1

## Discussion

In this study, we introduce a new open surgery dataset for gesture recognition, employing a multimodal approach with video data, hand pose, and tool pose. We utilize two models–MS-TCN++  and LTContext  –and find that an ensemble of all modalities yields the best results, highlighting the benefits of incorporating pose data.

As mentioned, capturing operating room (OR) videos presents numerous challenges, including variable camera positioning and difficult lighting conditions. It is difficult to predict how these factors might affect gesture recognition models that rely solely on video input. By employing pose data, we effectively decouple the problem. Assuming the pose estimator is robust under these conditions, the subsequent gesture recognition step is unaffected by the variability of the video recordings.

Our pose models are developed using large datasets, including synthetic data that enables training under simulated real-world conditions. In addition, temporal filtering can be applied to stabilize pose estimators, while rotation augmentations address changes in camera positioning. Further research is needed to assess the robustness of these models across diverse recording scenarios. In addition, future work should explore the development of online models, which are particularly valuable in surgical settings as they enable real-time monitoring and can promptly alert physicians to potential issues. Although the videos in our dataset are fairley long, the overall number of videos and unique gestures remains limited. Future efforts will therefore focus on expanding the dataset by including more videos and a broader range of gestures.

Furthermore, employing an ensemble model composed solely of the two pose estimators yielded results comparable to those obtained with video data alone. This finding suggests that video data could potentially be replaced by pose data, providing several advantages: videos may contain identifying information, and storing and analyzing them is resource-intensive. In contrast, pose data requires far fewer resources to store, transmit, and analyze. While combining video and pose data remains the superior approach, relying solely on pose data can be a viable alternative when necessary. Consequently, this study proposes a novel method for surgical data management and analysis.

## Appendix

See Tables 6, 7, 8 and 9
